# Combined PET-CT and MRI for response evaluation in patients with squamous cell anal carcinoma treated with curative-intent chemoradiotherapy

**DOI:** 10.1007/s00330-022-08648-z

**Published:** 2022-03-11

**Authors:** Pratik Adusumilli, Noha Elsayed, Stelios Theophanous, Robert Samuel, Rachel Cooper, Nathalie Casanova, Damien J. Tolan, Alexandra Gilbert, Andrew F. Scarsbrook

**Affiliations:** 1 Department of Radiology, Leeds Teaching Hospitals NHS Trust, Leeds, UK; 2 Department of Clinical Oncology, Leeds Teaching Hospitals NHS Trust, Leeds, UK; 3 Leeds Institute of Medical Research, Faculty of Medicine, University of Leeds, Leeds, UK; 4 Department of Nuclear Medicine, St James’s University Hospital, Level 1, Bexley Wing, Beckett Street, Leeds, West Yorkshire LS9 7TF UK

**Keywords:** Anus neoplasms, Carcinoma, squamous cell, Chemoradiotherapy, Magnetic resonance imaging, Positron-emission tomography

## Abstract

**Objectives:**

To assess the effectiveness of fluorine-18 fluorodeoxyglucose (FDG) positron-emission tomography-computed tomography (PET-CT) and magnetic resonance imaging (MRI) for response assessment post curative-intent chemoradiotherapy (CRT) in anal squamous cell carcinoma (ASCC).

**Methods:**

Consecutive ASCC patients treated with curative-intent CRT at a single centre between January 2018 and April 2020 were retrospectively identified. Clinical meta-data including progression-free survival (PFS) and overall survival (OS) outcomes were collated. Three radiologists evaluated PET-CT and MRI using qualitative response assessment criteria and agreed in consensus. Two-proportion *z* test was used to compare diagnostic performance metrics (sensitivity, specificity, positive predictive value (PPV), negative predictive value (NPV), accuracy). Kaplan-Meier analysis (Mantel-Cox log-rank) was performed.

**Results:**

MRI (accuracy 76%, PPV 44.8%, NPV 95.7%) and PET-CT (accuracy 69.3%, PPV 36.7%, NPV 91.1%) performance metrics were similar; when combined, there were statistically significant improvements (accuracy 94.7%, PPV 78.9%, NPV 100%). Kaplan-Meier analysis demonstrated significant differences in PFS between responders and non-responders at PET-CT (*p* = 0.007), MRI (*p* = 0.005), and consensus evaluation (*p* < 0.001). Cox regression analysis of PFS demonstrated a lower hazard ratio (HR) and narrower 95% confidence intervals for consensus findings (HR = 0.093, *p* < 0.001). Seventy-five patients, of which 52 (69.3%) were females, with median follow-up of 17.8 months (range 5–32.6) were included. Fifteen of the 75 (20%) had persistent anorectal and/or nodal disease after CRT. Three patients died, median time to death 6.2 months (range 5–18.3).

**Conclusion:**

Combined PET-CT and MRI response assessment post-CRT better predicts subsequent outcome than either modality alone. This could have valuable clinical benefits by guiding personalised risk-adapted patient follow-up.

**Key Points:**

*• MRI and PET-CT performance metrics for assessing response following chemoradiotherapy (CRT) in patients with anal squamous cell carcinoma (ASCC) were similar.*

*• Combined MRI and PET-CT treatment response assessment 3 months after CRT in patients with ASCC was demonstrated to be superior to either modality alone.*

*• A combined MRI and PET-CT assessment 3 months after CRT in patients with ASCC has the potential to improve accuracy and guide optimal patient management with a greater ability to predict outcome than either modality alone*

**Supplementary Information:**

The online version contains supplementary material available at 10.1007/s00330-022-08648-z.

## Introduction

Anal cancer is rare, accounting for 1.5% of digestive tract cancers with a worldwide incidence of 1 in 100,000 and rising prevalence in developed countries [1–3]. Ninety percent are of squamous cell histology (ASCC), with the most prevalent risk factor being human papillomavirus (HPV) [4–7]. Clinical staging is based on the American Joint Committee on Cancer (AJCC) TNM staging system [8]. Diagnosis and local staging are performed with a combination of physical examination, digital rectal examination, biopsy and pelvic magnetic resonance imaging (MRI). Fluorine-18 fluorodeoxyglucose (FDG) positron-emission tomography-computed tomography (PET-CT) is also recommended due to its high sensitivity in identifying nodal disease and distant metastases [9].

The mainstay of curative treatment for non-metastatic ASCC includes external-beam radiotherapy with concurrent mitomycin C and 5-fluorouracil or capecitabine [10]. A recent prospective, national cohort study using contemporary chemoradiotherapy (CRT) techniques reported 1-year overall survival (OS) rates of 94%, disease-free survival (DFS) of 84% and colostomy-free survival at 86% [11]. Five-year OS for all stages is around 60% [12]. Surgical treatment is largely reserved for salvage therapy in cases of CRT failure or recurrence. Consequently, post-CRT follow-up assessment, including high-quality imaging, is of key importance for early identification of residual or recurrent disease.

Post-CRT evaluation involves physical examination (inguinal lymph nodes and digital rectal examination (DRE)) and pelvic MRI 3 months after treatment, with subsequent follow-up guided by clinical and imaging findings (typically a repeat MRI at 6 months followed by CT scans at 1, 2 and 3 years) [13]. Currently, FDG PET-CT is not widely used for post-CRT ASCC treatment response evaluation. Studies have reported that post-treatment FDG PET-CT has a positive predictive value (PPV) of up to 85% and a very high negative predictive value (NPV) in the evaluation of local disease [14–19]. A recent systematic review highlighted the need for further work in evaluating the role of FDG PET-CT in the ASCC response assessment setting [20].

FDG PET-CT has the potential to bolster response assessment accuracy and help guide risk stratification at an earlier timepoint for increased surveillance, without the potential morbidity of biopsy and/or treatment escalation. It might also enable a de-escalation of imaging and clinical surveillance in patients who demonstrate complete metabolic response. This hypothesis is based on more established practice in other squamous cell tumours, such as head and neck and cervical cancer, where PET-CT assessment of treatment response has been widely used for several years with proven benefit in optimising patient management [21–26].

The aim of this retrospective study was to assess the effectiveness of FDG PET-CT in assessing ASCC treatment response 3 months post-CRT, compared to standard-of-care MRI, and to evaluate the combined efficacy of dual modality response evaluation for risk stratification.

## Methods

### Patient selection

Consecutive patients with histologically proven ASCC undergoing baseline and response assessment MRI and FDG PET-CT at a single large tertiary referral centre between January 2018 and April 2020 were identified retrospectively using a keyword search of the institutional Radiology Information System (RIS) (CRIS, Wellbeing Software). Only patients treated with curative-intent CRT using standardised departmental protocols were included. Patients received intensity-modulated radiotherapy with concurrent mitomycin C and 5-flurouracil or capecitabine. In general, patients with T1/T2 N0 disease received 50.4 gray (Gy) in 28 fractions and those with T3/T4 N0 or node-positive disease received 53.2 Gy in 28 fractions to gross tumour volume, using standard protocols. Exclusions were for patients with small surgically resected tumours, patients with metastatic disease not encompassable within the radiotherapy volume or those unfit for curative-intent CRT. The institutional Electronic Patient Record system (EPR) (PPM+) was utilised to obtain patient demographics, clinical history, staging and treatment details. The pertinent follow-up information included PFS and OS. PFS was defined as time from treatment completion to locoregional failure, new distant metastatic disease or ASCC-related death. OS was defined as time from CRT completion to death due to any cause.

Prospective consent for imaging data usage in research and service development projects was obtained from all patients at the time of imaging. Formal ethics committee approval was waived for this study which was considered by the institutional review board to represent evaluation of a routine clinical service.

### Imaging acquisition

All PET-CT studies were performed using standardised departmental protocols on Discovery 690 and 710 scanners (GE Healthcare). Both machines used iterative reconstruction, CT for attenuation correction and anatomical localisation, applied scatter and randoms correction. Image reconstruction and acquisition parameters are outlined in Supplemental Table 1. Serum blood glucose was routinely measured prior to imaging and if > 10 mmol/L scanning was not performed. Patients fasted for 6 h prior to intravenous injection of 4 MBq/kg of fluorine-18 FDG. Imaging was acquired from skull base to upper thighs 60 min following tracer injection. No iodinated contrast media was administered.

All MRI scans were performed on a 1.5-T scanner (Siemens Aera, Siemens Healthcare) using a phased array pelvic body coil. The following sequences were acquired: small field-of-view high-resolution sagittal, axial and coronal T2-weighted (T2W) sequences (slice thickness 3 mm); multi-shot turbo spin echo (TSE) T2W sequence (BLADE) (slice thickness 5 mm); axial diffusion-weighted image (DWI) sequences (b50, b700) and associated apparent diffusion coefficient (ADC) map (slice thickness 6 mm). Image reconstruction and acquisition parameters are outlined in Supplemental Table 2. All patients had a contrast-enhanced CT performed as part of initial staging which included a portal venous phase assessment of the liver to exclude metastases.

### Image analysis

MRI interpretation was undertaken using the institutional picture archiving and communication system (PACS) (Agfa IMPAX Version 5.6, AGFA Healthcare). PET-CT interpretation was undertaken on a multimodality workstation (Advantage Windows Version 3.5, GE Healthcare). Image analysis was performed by a radiologist with 3 years’ experience under the supervision of a dual-certified radiologist and nuclear medicine physician and a gastrointestinal radiologist with > 15 years’ experience of PET-CT and MRI respectively. All readers were blinded to clinical information prior to interpretation other than the diagnosis of anal cancer. As with routine clinical practice, the post-CRT imaging was compared with baseline studies.

Images were scored on a 5-point scale consisting of complete response (CR), partial response (PR), indeterminate (I), stable disease (SD) and disease progression (PD). Interpretation was applied on a per-patient basis. Discrepant findings were agreed in consensus.

### PET-CT interpretation criteria

Significantly reduced uptake with reduced tumour volume and resolution of nodal disease with metabolic activity less than mediastinal blood pool activity was categorised as CR; focal uptake greater than mediastinal pool but lower than uptake in the baseline study was categorised as PR; prominent physiological uptake within the anal canal potentially masking residual tumour activity was categorised I; unchanged activity was categorised SD; increased metabolic activity and/or increased tumour volume and/or new nodal disease or metastatic disease was categorised PD.

### MRI interpretation criteria

Tumour response at MRI was assessed on T2W sequences by evaluating morphological appearances and signal intensity within the anorectum and/or nodal disease and/or metastatic disease within the imaged volume. Complete resolution of the primary tumour mass and/or no residual intermediate T2W signal and/or resolution of nodal disease was considered CR; residual intermediate T2W signal intensity was considered PR; areas of abnormality that could not be attributed to residual disease or post-CRT-related changes were considered to be I; increased tumour volume and/or new nodal disease or metastatic disease was categorised PD.

DWI was assessed in conjunction with the corresponding ADC map. High signal on DWI sequences with associated low ADC in the original tumour indicated residual tumour and were considered PR, SD or PD depending on the size of the lesion as per RECIST 1.1 criteria [27].

### Combined PET-CT and MRI criteria

Concordant findings at both techniques were classified as such. Table 1 illustrates how the overall (combined) criteria were derived when there were divergent criteria at individual PET-CT or MRI assessment.

**Table 1:** Combined PET-CT and MRI interpretation criteria

		PET-CT				
		CR	PR	I	SD	PD
MRI	CR	CR	CR	CR	CR	PD
	PR	CR	PR	PR	PR	PD
	I	CR	PR	I	SD	PD
	SD	CR	PR	SD	SD	PD
	PD	PD	PD	PD	PD	PD

*CR* complete response, *PR* partial response, *I* indeterminate, *SD* stable disease, *PD* disease progression

### Clinical follow-up

Physical examination consisting of inguinal lymph node palpation and DRE was carried out 3 months post-CRT. When PET-CT and MRI imaging both demonstrated complete response, clinicians may omit a physical examination in the absence of any symptoms. Standard practice includes a further follow-up MRI and physical examination to be performed at 6 months post-treatment completion. On-going clinical follow-up, including physical examination, is 3-monthly for the first 2 years, 6-monthly years 3 and 4, and 12-monthly in the final year, supplemented by CT scans at 1, 2 and 3 years. True complete responses were defined as cases where locoregional control had been sustained over multiple follow-up evaluations. Evaluation comprised a composite outcome of clinical assessment (history and examination) and imaging follow-up assessment (as described). Additional imaging (e.g. at 9 or 12 months) was requested in equivocal cases, as recommended by the multidisciplinary team (MDT). Any equivocal cases during follow-up combine these assessment modalities to monitor for progression or resolution of changes, and biopsies are reserved for cases with concerning features.

### Statistical analysis

All statistical analyses were performed using SPSS Statistics 26 (IBM Corporation). Two-proportion *z* test was utilised to compare differences in sensitivity, specificity, PPV, NPV and accuracy. A two-tailed *p* value of < 0.05 was considered statistically significant. Time to event was measured from date of CRT completion. Kaplan-Meier survival analysis (Mantel-Cox log-rank) Cox regression hazard ratios were calculated and used to assess PFS and OS. Survival curves for OS and PFS were constructed using the Kaplan-Meier method (and compared with the Mantel-Cox log-rank test). Hazard ratios (HR) were calculated using the Cox proportional hazards model.

## Results

### Patient cohort and follow-up

Seventy-five patients were included; 52 (69.3%) were female and 23 (30.7%) were male with a median age of 62 years (range 35–85 years). The median time interval between end of treatment and MRI was 100 days (range 42–219 days) and 96 days for PET-CT (range 37–255 days). Differences in imaging study scheduling were related to scanner availability, interval unplanned medical care and more recently the COVID-19 pandemic. All patients were followed up either until death or 31^st^ December 2020 with a median follow-up time of 540 days (range 151–993 days). Table 2 outlines patient characteristics in more detail.

**Table 2:** Patient cohort synopsis

Total patients	75
Male	23
Female	52
Median age at start of treatment (years)	62
Range	35–85
Stage	
T1	4
T2	36
T3	22
T4	13
Nodal stage at baseline	
N0	33
N1a	29
N1b	2
N1c	13
Metastatic stage at baseline	
M0	72
M1	3
Median primary tumour SUVmax	13.2
Range	4.2–33.4
Treatment failures*	
No	60
Yes	15
Median time from end of treatment to response assessment MRI (days)	100
Range	42–219
Median time from end of treatment to response assessment PET-CT (days)	96
Range	37–255
Deaths	3
Progression	15
Median follow-up period (days)	540
Range	151–993

*Breakdown of disease failure site provided in text

### Overall and progression-free survival

Fifteen of the 75 (20%) patients had persistent anorectal and/or nodal disease after CRT (defined as ‘clinical outcome’). This included 8 patients with residual anorectal disease, 4 with interim progression of the primary tumour and 1 with persistent anorectal and inguinal nodal disease. One patient developed locoregional metastatic disease, and another had new systemic metastatic disease identified on response assessment imaging. Three patients with residual disease (20%) died due to disease progression during the follow-up period, including one individual who progressed through treatment and died shortly after completion of CRT. PFS and OS rates were 79.3% and 95.0% respectively. Median time to progression was 165 days (range 181–557 days), and median time to death was 189 days (range 19–517 days).

### Imaging response assessment

#### Image stratification

To allow comparison with clinical outcome, imaging studies were stratified into complete response and residual disease categories, the latter encompassing partial response (PR), indeterminate (I), stable disease (SD) or disease progression (PD) classifications.

#### Image classification

When considered as stand-alone techniques, PET-CT and MRI demonstrated complete response in 45 patients (60.0%) and 46 patients (61.3%) respectively. MRI false-negative rate was slightly lower (2 patients, 2.7%) than PET-CT (4 patients, 5.3%) as was the false-positive rate at MRI (16 patients, 21.3%) compared to PET-CT (19 patients, 25.3%). When both studies were considered in combination, accuracy of classification compared to physical examination significantly improved with 56 (of 60) complete responders correctly identified. The false-positive rate reduced significantly (4 patients, 5.3%), and there were no false-negative interpretations. Performance metrics of individual and combined imaging assessments are detailed in Table 3.

**Table 3:** Performance metrics for FDG PET-CT, MRI and combined assessment of treatment response compared to clinical outcome

Response	Clinical outcome	FDG PET-CT	MRI	Consensus
Responders (CR)	60 (80%)	45 (60%)	46 (61.3%)	56 (74.7%)
Residual disease (PR + SD + I + PD)	15 (20%)	30 (40%)	29 (38.7%)	19 (25.3%)
False-positive findings	–	19	16	4
False-negative findings	–	4	2	0
True-positive findings	–	11	13	15
True-negative findings	–	41	44	56
Sensitivity	–	73.3%	86.7%	100%
Specificity	–	68.3%	73.3%	93.3%
Positive predictive value	–	36.7%	44.8%	78.9%
Negative predictive value	–	91.1%	95.7%	100%
Accuracy	–	69.3%	76%	94.7%

*CR* complete response, *PR* partial response, *I* indeterminate, *SD* stable disease, *PD* disease progression

Two-proportional *z* test comparison of PET-CT, MRI and combined response assessment performance metrics is documented in Supplemental Table 3. MRI had a higher sensitivity than PET-CT (*p* = 0.04, 86.7 vs 73.3%). Most performance metrics showed statistically significant improvements when a combined approach was used.

Overall, 49 patients (65.3%) had concordant response classification at MRI and PET-CT (Table 4). In the 26 discordant studies, MRI classification aligned with physical examination in 16 cases (64.3%). In 13 of these cases (81.2%), PET-CT classification was discordant due to residual anal canal activity in the absence of MRI signal change either due to prominent physiological uptake or post-treatment-related inflammation. PET-CT classification matched physical examination in 9 patients (32.1%); in this sub-group, 6 patients had residual T2W signal change at the site of the ASCC. There was only 1 case (3.6%) where both MRI and PET-CT classification did not correlate with physical examination, there was a complete local response, but imaging demonstrated a new bone metastasis. Twelve patients (46.2%) had a less marked response on PET-CT than on MRI. Discordance with physical examination reduced to only 5 patients (6.7%) when PET-CT and MRI were considered in consensus. Of note, 12 patients (16%) who had residual T2W signal change on MRI but with complete response on PET-CT and at physical examination could have avoided further MRI assessment at 6 months using a combined PET-CT and MRI response to stratify further management.

**Table 4:** Comparison between PET-CT and MRI response classifiers

	FDG PET-CT					
MRI	CR	PR	I	SD	PD	Total
CR	*34*	9	2	1	0	46
PR	11	*13*	0	0	0	24
I	0	0	*1*	0	0	1
SD	0	0	0	*0*	0	0
PD	0	2	0	1	*1*	4
Total	45	24	3	2	1	75

*CR* complete response, *PR* partial response, *I* indeterminate, *SD* stable disease, *PD* disease progression

The highlighted data (italics) emphasises exact response concordance between the two modalities

**Fig. 1:**
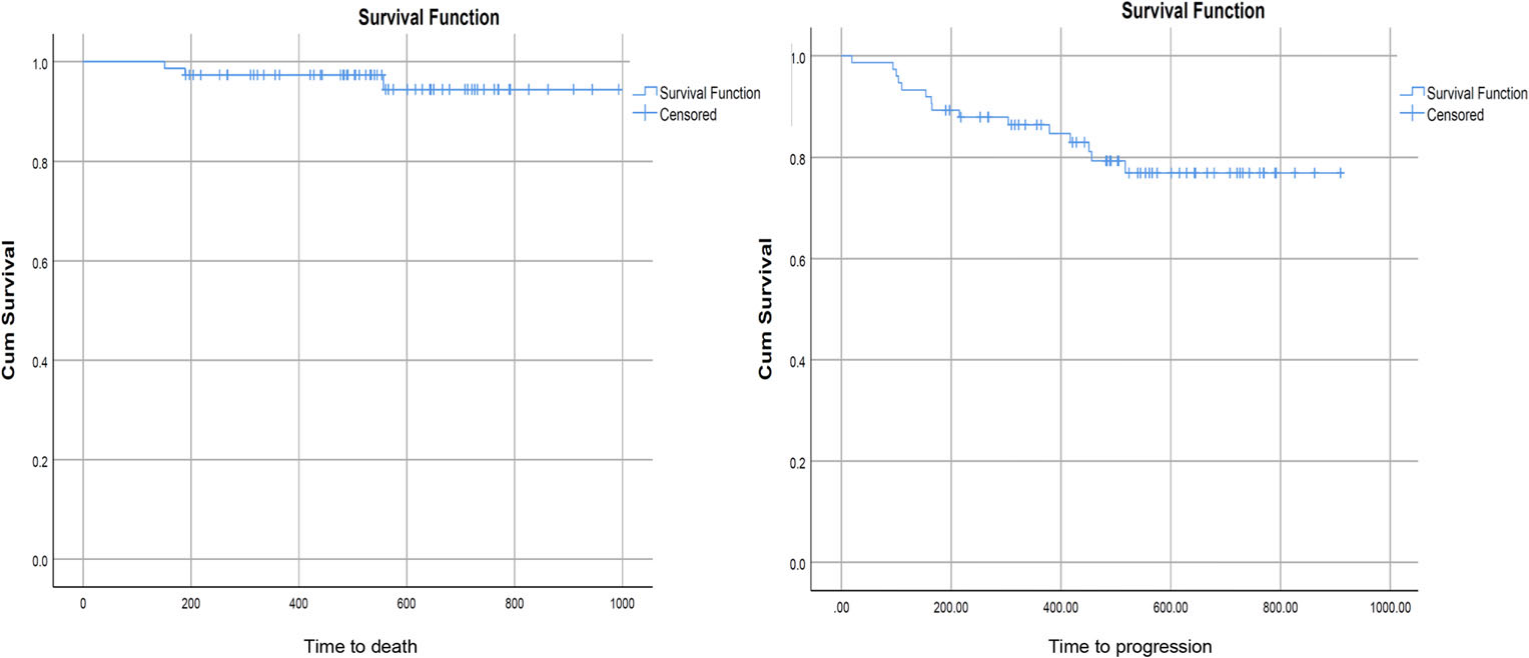
Kaplan-Meier graphs of progression-free (right) and overall survival (left)

**Fig. 2:**
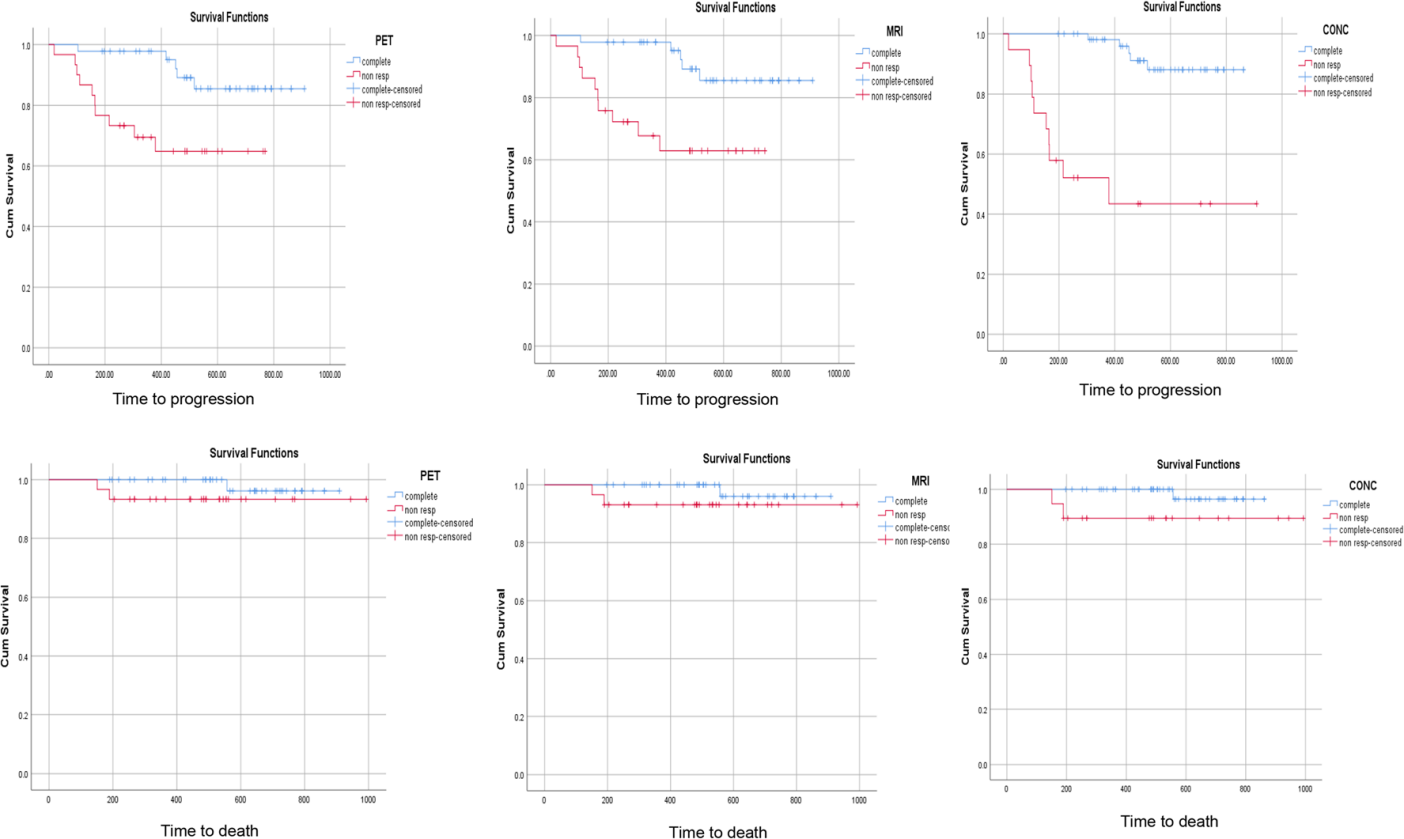
Kaplan-Meier graphs of time to progression and time to death for PET-CT, MRI and consensus assessment

**Fig. 3:**
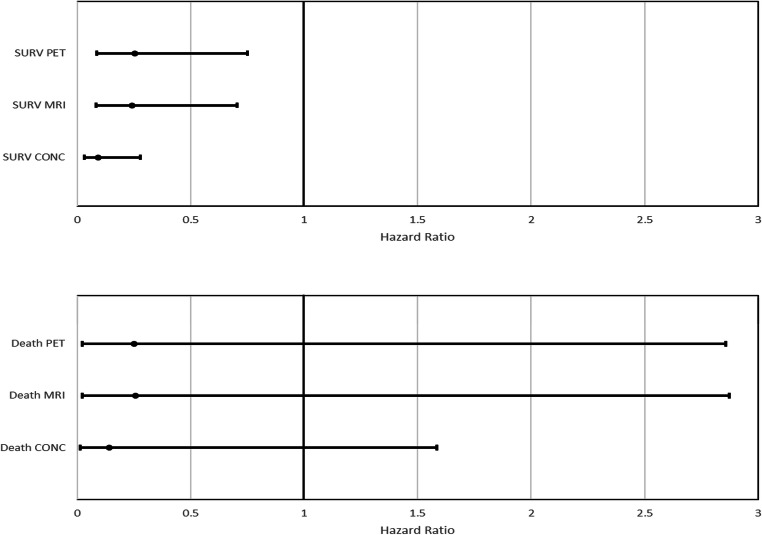
Forest plots showing the hazard ratios and 95% confidence intervals for progression-free (top row) and overall survival (bottom row)

Kaplan-Meier analysis (Fig. 1) demonstrated statistically significant differences in PFS between responders (mean 863 days ± 31) and non-responders at PET-CT (mean 562 days ± 54) (*p* = 0.007), MRI (*p* = 0.005) (mean 836 days ± 30 versus mean 535 days ± 53) and consensus read (*p* < 0.001) (mean 811 days ± 21 versus mean 488 days ± 91). There were no significant differences in OS, likely related to the small event rate (Fig. 2, Table 5). Cox regression analysis of PFS demonstrated a lower hazard ratio (HR) and narrower 95% confidence intervals for consensus findings: PET-CT (HR = 0.255, *p* = 0.013), MRI (HR = 0.240, *p* = 0.009) and consensus (HR = 0.093, *p* < 0.001). HR for consensus findings in OS was lower than that for PET-CT and MRI alone, but was not statistically significant, likely related to the small number of deaths in the cohort (Table 5, Fig. 3).

**Table 5:** (a) Log-rank (Mantel-Cox) and (b) Cox regression survival analyses

**(a)**				
	Chi-square	df		*p* value
Progression-free survival analysis				
PET-CT	7.160	1.000		**0.007**
MRI	7.920	1.000		**0.005**
Consensus	27.247	1.000		**0.000**
Overall survival analysis				
PET-CT	1.438	1.000		0.231
MRI	1.411	1.000		0.235
Consensus	3.396	1.000		0.065
**(b)**				
	Hazard ratio	95% CI lower	95% CI upper	*p* value
Progression-free survival analysis				
PET-CT	0.255	0.087	0.749	**0.013**
MRI	0.240	0.082	0.705	**0.009**
Consensus	0.093	0.031	0.277	**0.000**
Overall survival analysis				
PET-CT	0.250	0.022	2.857	0.265
MRI	0.256	0.023	2.874	0.269
Consensus	0.142	0.013	1.584	0.113

*CI* confidence interval, *df* degrees of freedom

The bold values emphasise statistically significant *p* values

## Discussion

In this retrospective series, it has been demonstrated that a response assessment protocol combining FDG PET-CT and MRI can identify patients with CR post-CRT more accurately than either technique alone. Most importantly, CR on combined imaging was a powerful predictor of sustained complete clinical response.

FDG PET-CT is well-established for evaluating the efficacy of CRT in patients with SCC of the head and neck and cervix. Growing evidence suggests FDG PET-CT may have similar utility in patients with ASCC although its use is not widespread [17, 28–34]. MRI is widely used to assess response following CRT in ASCC although the evidence supporting this consists of a small number of single-centre retrospective studies only [35–38]. The utility of combined FDG PET-CT and MRI for post-CRT response assessment in ASCC patients has not been reported.

In this study, MRI and PET-CT response assessment performance metrics for evaluation of local disease and loco regional metastases were broadly similar except that MRI demonstrated a higher sensitivity (86.7% vs 73.3%). FDG PET-CT had a PPV and NPV of 36.7% and 91% respectively. The PPV is lower than most published series (median 67%, range 25–85%) which performed imaging at variable timepoints after CRT (Supplemental Table 4) [14–19, 29, 39]. Prominent physiological tracer activity or post-treatment inflammatory uptake within the anal cancer can hamper accurate assessment, and an optimal imaging timepoint has yet to be established. NPV is towards the upper end of the range of reported values (median 96.4%, range 67–100%) [14, 16, 18, 19, 29, 39]. MRI had a PPV and NPV of 44.8% and 95.7%, respectively, which are comparable to the only published performance metrics of MRI in this setting (PPV 42–60%, NPV 94–100%) (Supplemental Table 4) [37, 38]. These two single-centre studies evaluated 39 and 74 patients respectively, the larger study utilising a tumour regression grading system to allow reproducible assessment of local tumour response on MRI [37]. Other MRI studies evaluated small groups of patients with ASCC immediately, 6–8 weeks and 6 months after CRT and found earlier timepoint imaging was unhelpful due to post-treatment inflammation [36, 37].

**Fig. 4:**
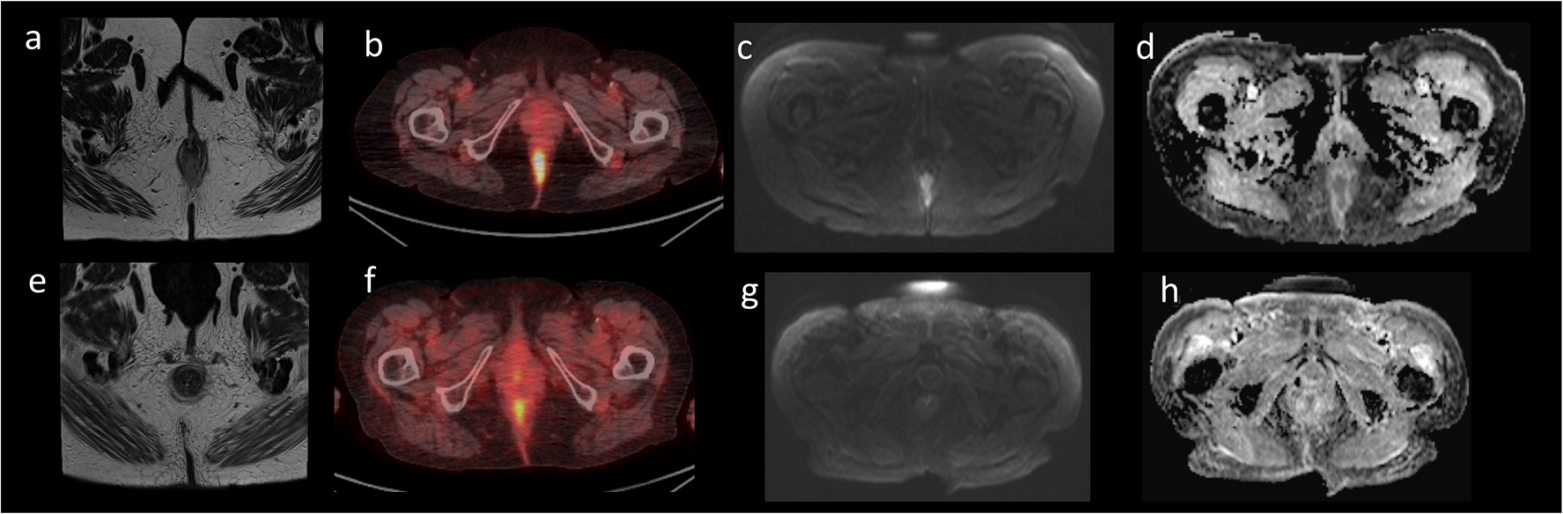
84-year-old female. Axial images. MRI response assessment: complete, PET-CT response assessment: partial, combined response assessment: complete. **a** Pre-treatment T2W MRI demonstrates tumour involvement between the 4 o’clock and 6 o’clock position. **b** Pre-treatment PET-CT demonstrates avid FDG uptake within the anal canal. **c** DWI demonstrated diffusion restriction (high signal). **d** The ADC confirms true restriction (low signal). **e** Post-treatment T2W MRI demonstrates replacement of the primary tumour with a small area of fibrotic tissue. **f** Post-treatment PET-CT demonstrates residual focus of moderate FDG uptake which may either represent residual disease or post-treatment inflammatory change. **g** The DWI sequence does not demonstrate restriction. **h** The lack of diffusion restriction is confirmed by the ADC map. Overall, due to the lack of diffusion restriction and evidence of fibrotic tissue on the T2W MRI, the patient was considered as a complete responder. The focus of FDG uptake on the response assessment FDG PET-CT was most likely inflammatory in nature

**Fig. 5:**
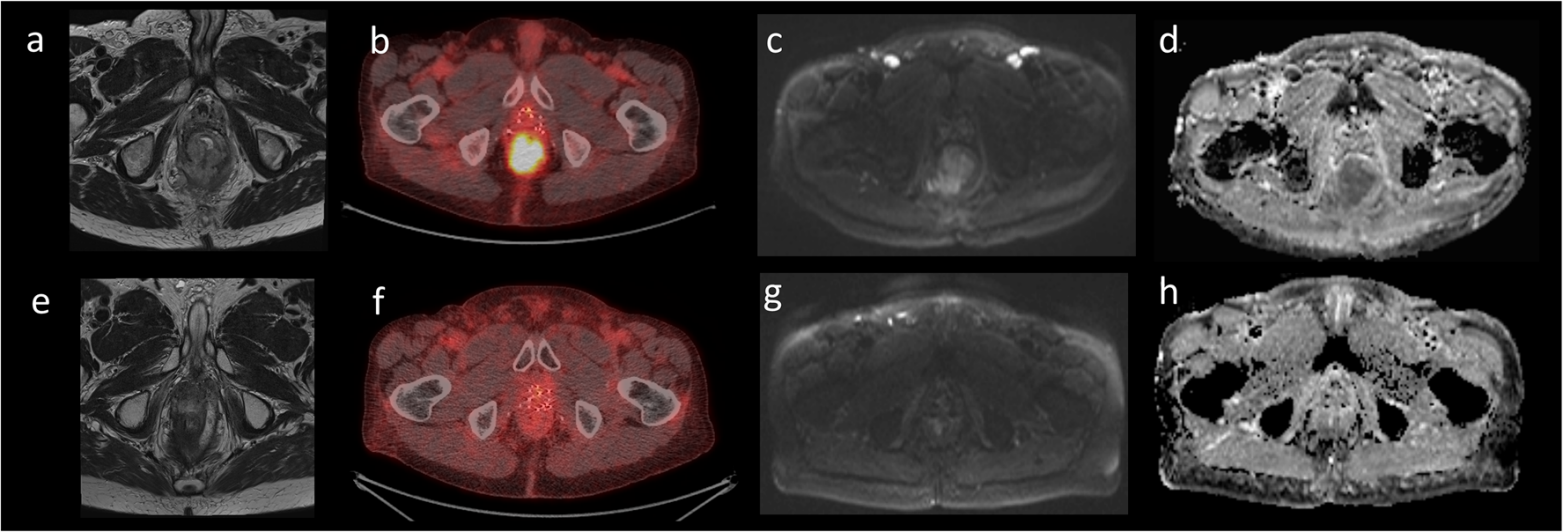
71-year-old male, brachytherapy seeds within the prostate. Axial images. MRI response assessment: partial, PET-CT response assessment: complete, combined response assessment: complete. **a** Pre-treatment T2W MRI demonstrates an almost annular locally advanced tumour centred at the anorectal junction extending into the perineum and rectum. **b** Pre-treatment PET-CT demonstrates a large metabolically active mass involving the anal canal and lower rectum; bilateral groin lymph nodes demonstrate moderate uptake and are likely involved. **c** DWI demonstrates diffusion restriction at the site of the tumour as well as inguinal node involvement (high signal). **d** The ADC confirms true restriction (low signal). **e** Post-treatment T2W MRI demonstrates reduced tumour volume which is largely fibrotic. Focal bowel wall oedema was present which may represent residual tumour or post-treatment inflammatory changes. No residual nodes are demonstrated. **f** Post-treatment PET-CT demonstrates no residual FDG avid disease at the site of the previous tumour and nodes. **g** The DWI sequence does not demonstrate restriction. **h** The lack of diffusion restriction is confirmed by the ADC map. Overall, due to the lack of residual FDG avid disease, the patient was considered as a complete responder. The focal bowel wall oedema on the response assessment MRI was most likely inflammatory in nature.

When PET-CT and MRI findings were considered in combination, a tangible benefit was demonstrated with statistically significant improvements in false positive, true negative, sensitivity, specificity, PPV and accuracy metrics compared with MRI interpretation alone (Table 3). Figures 4 and 5 demonstrate cases where combined PET-CT and MRI evaluation provided extra benefit. PPV and NPV for combined PET-CT and MRI assessment was 78.9% and 100% respectively. This has potentially important clinical implications by facilitating personalised care for patients with more streamlined follow-up in those with CR. This would allow cost savings (reduced visits/imaging) and additional patient benefits in terms of the reduced emotional burden of cancer survival and follow-up. In this study, stratified follow-up using a combined PET-CT and MRI approach could have potentially avoided further imaging and/or additional digital rectal examinations in 16% of patients.

Similarly, this was reflected in PFS prediction; both imaging techniques individually demonstrated similar HRs and 95% CI, with a much lower HR and narrower 95% CI for combined findings. Due to the low number of deaths in this cohort and short follow-up, OS could not be analysed to the same degree of confidence. However, combined findings demonstrated a lower HR and narrower 95% CI.

Several single-centre studies in anal and cervical cancer have reported OS rates in patients with CR on PET-CT of around 90% at 5 years and 95% at 2 years, with 95–100% NPV [25, 29, 38] and a very low rate of asymptomatic recurrence (1.6% in cervical cancer patients) [40]. The ability to reliably predict lack of recurrence using imaging could allow personalisation of follow-up care with a focus on detection and management of late toxicity. The high cure rate, low prevalence of asymptomatic recurrence and high toxicity rates make ASCC an ideal target for designing a remote symptom-led follow-up pathway. However, it remains important to have appropriate safeguards so that recurrence is not missed in a small minority of these low-risk patients. Following curative CRT for head and neck cancer (a similar cancer biologically to cervical and anal cancer), PET-CT response was successfully used as a method to stratify follow-up, reducing costs by AUD$5012 per patient (£2738) over 5-years follow-up [41]. Importantly there were no differences in time-to-recurrence detection or impact on ability to radically treat recurrences between a historical and reduced follow-up cohort.

There are study limitations including the retrospective single-centre nature and relatively small cohort size, which reflects the low incidence of ASCC. Additionally, median follow-up was 1.4 years and normally patients would be followed for up to 5 years. However, less than 1% of relapses occur after 3 years and the majority of locoregional failures occur within the first 18 months to 2 years following treatment and therefore the trends seen in this study are promising [10]. While clinical assessment (history and examination) is an imperfect reference standard, most local recurrences are either symptomatic or palpable and our clinical and imaging follow-up schedule is in line with the recently published ESMO guidelines [13]. Due to the COVID-19 pandemic, a small proportion of patients who completed treatment in 2020 had delayed response assessments.

Regarding use of contrast-enhanced sequences with MRI, there is variation in practice. Some authors have shown a role for gadolinium contrast evaluation. This is not standard of care in the UK and its adoption is variable internationally [38, 42].

The potential benefits of a multimodality imaging approach for assessing response to CRT in ASCC have been demonstrated. Prospective evaluation in combination with risk-adapted patient follow-up warrants further investigation.

## Conclusion

Combined PET-CT and MRI response assessment post-CRT was a better predictor of subsequent outcome than either modality alone. This could have valuable clinical benefits by guiding personalised risk-adapted patient follow-up.

## Supplementary Information

ESM 1 (DOCX 34 kb)

## Abbreviations

ADC: Apparent diffusion coefficient
AJCC: American Joint Committee on Cancer
ASCC: Anal squamous cell carcinoma
CR: Complete response
CRT: Chemoradiotherapy
DFS: Disease-free survival
DWI: Diffusion-weighted imaging
EPR: Electronic patient record
FDG: Fluorine-18 fluorodeoxyglucose
Gy: Gray
HPV: Human papillomavirus
HR: Hazard ratio
I: Indeterminate
MRI: Magnetic resonance imaging
NPV: Negative predictive value
OS: Overall survival
PACS: Picture archiving and communication system
PD: Disease progression
PET-CT: Positron-emission tomography-computed tomography
PR: Partial response
RIS: Radiology information system
SD: Stable disease
T2W: T2 weightedm
TSE: Turbo spin echo

## Declarations

### Guarantor:

The scientific guarantor of this publication is Professor Andrew F. Scarsbrook.

### Statistics and Biometry:

No complex statistical methods were necessary for this paper.

### Methodology

• retrospective

• observational

• performed at one institution
