# Supplementary material for: Combined PET-CT and MRI for response evaluation in patients with squamous cell anal carcinoma treated with curative-intent chemoradiotherapy
Source: Eur Radiol. 2022 Mar 11;32(8):5086–96. doi: 10.1007/s00330-022-08648-z (PMC8913212; doi:10.1007/s00330-022-08648-z)
Supplement: Supplementary file 1 — (DOCX 34 kb) [file 330_2022_8648_MOESM1_ESM.docx]

**Supplemental Material**

| **Scanner** | **Reconstruction** | **Scatter correction** | **Randoms correction** | **Matrix** | **Voxel size (mm)** |
| --- | --- | --- | --- | --- | --- |
| GE Healthcare Discovery 690 | VPFX | Model based | Singles | 192 | 3.65 x 3.65 x 3.27 |
| GE Healthcare Discovery 710 | VPFX | Model based | Singles | 192 | 3.65 x 3.65 x 3.27 |

**Supplemental Table 1: PET-CT image reconstruction and acquisition parameters**

Key : VPFX = Time of Flight Ordered Subset Expectation Maximization

| **Sequence** | | | | | | |
| --- | --- | --- | --- | --- | --- | --- |
|  | T2W | T2W | T2W | TSE T2W (BLADE) | DWI (b50, b700) | ADC map |
| **Plane** | Axial | Coronal | Sagittal | Axial | Axial | Axial |
| **Repetition time (ms)** | 4340 | 4340 | 6810 | 6790 | 5400 | 5400 |
| **Echo Time (ms)** | 96 | 96 | 93 | 83 | 74 | 74 |
| **Echo Train** | 11 | 11 | 15 | 19 | 61 | 61 |
| **Bandwidth (Hz/pixel)** | 120 | 120 | 150 | 260 | 1630 | 1630 |
| **Field of view (mm)** | 220x220 | 220x220 | 220x220 | 300x300 | 370x399 | 370x399 |
| **Slice Thickness (mm)** | 3 | 3 | 3 | 5 | 6 | 6 |
| **Slice Gap (mm)** | 3.3 | 3.3 | 3.6 | 5.5 | 7.2 | 7.2 |
| **Base Matrix** | 384x346 | 384x346 | 320x320 | 320x320 | 192x142 | 192x142 |
| **Pixel Size (mm)** | 0.57x0.57 | 0.60x0.60 | 0.69x0.69 | 0.94x0.94 | 2.1x2.1 | 2.1x2.1 |

**Supplemental Table 2: MRI acquisition parameters**

Key : ADC = Apparent diffusion coefficient, DWI = Diffusion weighted imaging, TSE = Turbo Spin Echo, T2w = T2 weighted

| **MRI vs PET-CT** | **Z value** | **P value** |  |  |  |  |  |  | | |
| --- | --- | --- | --- | --- | --- | --- | --- | --- | --- | --- |
|  |  |  |  |  |  |  |  |  | | |
| False Positive Findings | 0.5791 | 0.56192 |  |  | | | | | |  |
| False Negative Findings | 0.8333 | 0.40654 |  |  | | | | | |  |
| True Positive Findings | -0.4454 | 0.65272 |  |  | | | | | |  |
| True Negative Findings | -0.4943 | 0.62414 |  |  | | | | | |  |
| **Sensitivity** | **-2.0514** | **0.04036** |  |  | | | | | |  |
| Specificity | -0.6734 | 0.50286 |  |  | | | | | |  |
| Positive Predictive Value | -1.0095 | 0.3125 |  |  | | | | | |  |
| Negative Predictive Value | -1.1346 | 0.25848 |  |  | | | | | |  |
| Accuracy | -0.9204 | 0.35758 |  |  | | | | | |  |
|  |  |  |  |  |  |  |  |  | | |
| **MRI vs consensus** |  |  |  |  |  |  |  |  | | |
|  |  |  |  |  |  |  |  |  | | |
| **False Positive Findings** | **2.8823.** | **0.00398** |  |  | | | | | |  |
| False Negative Findings | 1.4237. | 0.1556 |  |  | | | | | |  |
| True Positive Findings | -0.4191 | 0.67448 |  |  | | | | | |  |
| **True Negative Findings** | **-2.0785** | **0.03752** |  |  | | | | | |  |
| **Sensitivity** | **-3.2689** | **0.00108** |  |  | | | | | |  |
| **Specificity** | **-3.2837** | **0.00104** |  |  | | | | | |  |
| **Positive Predictive Value** | **-4.2989** | **< .00001** |  |  | | | | | |  |
| Negative Predictive Value | -1.8155 | 0.06876 |  |  | | | | | |  |
| **Accuracy** | **-4.0486** | **< .00001** |  |  | | | | | |  |
|  |  |  |  |  |  |  |  |  | | |
|  |  |  |  |  |  |  |  |  | | |
| **PET-CT vs consensus** |  |  |  |  |  |  |  |  | | |
|  |  |  |  |  |  |  |  |  | | |
| **False Positive Findings** | **3.3992** | **0.00068** |  | | | | | |  |  |
| **False Negative Findings** | **2.0272** | **0.04236** |  | | | | | |  |  |
| True Positive Findings | -0.8628 | 0.38978 |  | | | | | |  |  |
| **True Negative Findings** | **-2.5622** | **0.01046** |  | | | | | |  |  |
| **Sensitivity** | **-4.8073** | **< .00001** |  | | | | | |  |  |
| **Specificity** | **-3.8869** | **0.0001** |  | | | | | |  |  |
| **Positive Predictive Value** | **-5.2325** | **< .00001** |  | | | | | |  |  |
| **Negative Predictive Value** | **-2.6431** | **0.0083** |  |  | | | | | |  |
| **Accuracy** | **-3.2384** | **0.0012** |  |  | | | | | |  |

**Supplemental Table 3**: Two proportional z-test comparison of MRI, PET-CT and combined response assessment performance metrics

| **Modality** | **Author/Publication Year**  **(reference number)** | **Cohort size** | **Imaging timepoint (median/range)** | **Follow-up duration** | **PPV** | **NPV** |
| --- | --- | --- | --- | --- | --- | --- |
| **PET-CT** | Trautman et al 2005 [16] | 18 | 1 month | 18 months minimum | 25% | 67% |
|  | Nguyen et al 2008 [15] | 25 | 3.9 months median (range 2.1 – 6.4) | 25 months median (range 1 – 86) | 40% | ----- |
|  | Schwarz et al 2008 [17] | 53 | 2 months median (range 0.9 – 5.4) | 26 months mean (range 5 – 68) | 67% | ----- |
|  | Day et al 2011 [31] | 34 | 2.4 months median (range 1.3 – 4.6) | 60 months median (range 20.4 – 109.2) | ----- | ----- |
|  | Vercellino et al 2011 [14] | 10 | 3.5 months median (range 1 – 5.6) | 13 months median (range 4 – 44) | 78% | 94% |
|  | Mistrangelo et al 2012 [39] | 43  40 | 1 month  3 months | 20.3 months median (range 1 – 60) | 43%  67% | 97.4%  100% |
|  | Goldman et al 2016 [18] | 148 | 2.9 months mean (range 1.2 – 5.8) | 34 months median (range 5 – 89) | 85% | 92.9% |
|  | Teagle et al 2016 [29] | 35 | Variable (majority 4-9 months post treatment) | 32 months median (range 1 – 104) | 71% | 100% |
|  | Houard et al 2017 [19] | 87 | 4 months median (range 1 – 8) | 25 months median (range 8 – 76.9) | 72% | 96.4% |
| **MRI** | Koh et al 2008 [35] | 15 | Immediate    6 months | Not reported | ----- | ----- |
|  | Goh et al 2010 [36] | 35 | 6-8 weeks | 33.5 months median (range 13.1 – 58.8) | ----- | ----- |
|  | Jederán et al (2015) [37] |  | 1.6 months median (range 0.7 – 3.7) | 12 months | 42% | 100% |
|  | Kochhar el al 2017 [38] | 74 | 3 months  6 months | 52 months median (range 9 – 72) | 60%  100% | 94%  96% |

**Supplemental Table 4**: Published studies evaluating FDG PET-CT or MRI for response assessment in ASCC
